# Sustainable development of copper matrix hybrid composites using waste stainless steel chips: a physical and tribological investigation

**DOI:** 10.1038/s41598-026-42090-1

**Published:** 2026-03-06

**Authors:** Manvandra Kumar Singh, Gopal Ji, Vineet Kumar, Ashwani Sharma, Uma Shankar, Pramod Kumar, Rajeev Nayan Gupta, Rohit Kumar Singh Gautam, Manish Kumar, Jitendra Kumar Katiyar

**Affiliations:** 1 Department of Mechanical Engineering, Graphic Era (Deemed to Be University), Dehradun, Uttarakhand 248002 India; 2 Department of Physics, Indian Institute of Technology (BHU) Varanasi, Varanasi, Uttar Pradesh 221005 India; 3 Micro Advanced Research Centre, Micro Labs Limited, Bangalore, Karnataka 560068 India; 4 Chhatrapati Shahu Ji Maharaj University, Kanpur, Uttar Pradesh 208024 India; 5 Department of Mechanical Engineering, National Institute of Technology Silchar, Silchar, Assam 766002 India; 6 Deptarment of Mechanical Engineering, Teerthanker Mahaveer University, Moradabad, Uttar Pradesh 244001 India; 7 Department of Physics, ARSD College, University of Delhi, Dhaula Kuan, New Delhi 110021 India; 8 Manipal Institute of Technology, Manipal Academy of Higher Education, Manipal, India

**Keywords:** Tribology, Hybrid composites, Sustainable composite, Waste metallic chips, Mechanical properties, Engineering, Materials science

## Abstract

Stainless steel is widely used in manufacturing industries, and its machining generates large quantities of metallic chips that are typically discarded as waste. In view of this, the present study aims to fabricate copper-based hybrid composites using waste stainless steel chips (WSSCs) via the stir-casting process. Four hybrid composites were produced by reinforcing the copper matrix with WSSCs, tungsten carbide (WC), and chromium (Cr). These composites were labelled HC-WSSC1, HC-WSSC2, HC-WSSC3, and HC-WSSC4, corresponding to 1, 2, 3, and 4 wt% WSSCs, respectively, while maintaining constant proportions of WC and Cr. Microstructural analysis confirmed a fair distribution of the reinforcement phases within the copper matrix. The measured density of the fabricated hybrid composites was lower than that of pure copper and decreased with increasing WSSC content. In contrast, Brinell hardness values increased progressively with higher WSSC reinforcement. Tribological performance, including friction and wear behaviour, was systematically evaluated under dry sliding conditions using a pin-on-disk tribometer. The results demonstrated that the hybrid composites possess superior wear resistance compared to the copper matrix, with further improvement observed at higher WSSC contents. Additionally, the worn surfaces were investigated using atomic force microscopy (AFM) and scanning electron microscopy (SEM) to reveal the dominant mechanisms of wear.

## Introduction

Copper, prized for its excellent electrical and thermal behaviour, is extensively used in industries spanning electrical contacts, heat exchangers, and automotive components. However, its inherently low hardness and poor wear resistance restrict its application in high-friction environments^1–4^. To address these deficiencies, researchers have long turned to copper metal matrix composites/hybrid composites, where copper serves as the ductile matrix for reinforcing particles. Copper metal matrix composites/hybrid composites combine the toughness and conductivity of copper with the enhanced mechanical and tribological properties provided by reinforcements. Particle-type reinforcements, including SiC, Al_2_O_3_, TiC, B_4_C, BN, TiC, WC, graphite, and hybrid mixtures, have been demonstrated to boost hardness, strength, and wear resistance^5–10^. Research consistently shows that adding hard ceramic particles leads to higher hardness and reduced wear, often nearing an optimum reinforcement fraction beyond which adverse effects may arise^11–14^. The sustainability imperative in materials science encourages repurposing industrial waste, for example, metallic chips from machining processes, as cost-effective reinforcements. This not only reduces environmental burden but also exploits reinforcement-like characteristics of hard metal debris. Aluminum-based metal matrix composites reinforced with waste steel chips have shown significant improvements in hardness and wear resistance when compared to traditional reinforcement materials^15–17^. Similarly, copper matrix composites infused with machined chips of steel have delivered comparable benefits in mechanical properties, wear performance, and corrosion resistance^4,18–20^. Existing literature highlights successes using steel chips in copper matrices, but explorations, especially involving waste metallic stainless-steel chips, remain scant. Stainless steel chips bring unique attributes to their higher hardness, corrosion resistance, and prevalent availability from machining operations, which could yield a distinct enhancement in wear behavior. Furthermore, the hybrid concept leveraging both lubricity and corrosion resistant reinforcements suggests promising avenues. However, combining stainless steel chips with other lubricating or ceramic additives in copper matrices has not been adequately studied. Some other researchers have also developed the similar kind of composite materials using different techniques and investigated the various properties such as mechanical, microstructural, and tribological etc. They observed that the studied properties of the developed composites were much better as compared to its matrix and found suitable for various applications^21–25^.

In this work, we propose and investigate hybrid copper metal matrix composites infused with waste chips of metallic stainless steel, tungsten carbide, and chromium fabricated by the stir-casting technique. This research is novel due to some points: 1-recycling industrial waste while positively impacting composite performance. 2-evaluating the tribological characteristics of stainless-steel chip reinforced copper composites, an unexplored reinforcement combination. 3-examining friction and wear responses, aiming to balance enhanced wear resistance, friction coefficient reduction, and potentially improved corrosion resilience. 4-systematically characterize and compare hardness, microstructure, and tribological responses under dry sliding conditions. 5-elucidate the prevailing friction and wear mechanisms in relation to chip content and distribution.

Adopting waste stainless steel chips as reinforcements aligns with circular economy goals, offering an economically beneficial path to high performance hybrid composites^26^. Anticipated improvements in wear resistance could render these materials suitable for electrical sliding contacts, bushings, bearings, and corrosion exposed components. Understanding the interplay of chip content, dispersion, and wear mechanisms also enriches fundamental insights into hybrid composite behavior.

The novel contribution of using waste stainless steel chips (WSSCs) in copper matrix hybrid composites lies in transforming machining scrap into value-added reinforcement for sustainable material development. Incorporating waste stainless steel chips into a copper matrix enhances mechanical strength, hardness, and wear resistance through effective load transfer and crack deflection, while largely retaining copper’s thermal and electrical conductivity. Hybridization with secondary reinforcements e.g., tungsten carbide (WC), chromium (Cr) further tailors multifunctional performance. This approach supports circular economy principles by reducing industrial waste and material cost. These composites are suitable for applications such as electrical contacts, bushings, bearings, heat sinks, and wear-resistant conductive components in automotive and power systems.

The novelty of the WSSCs + WC + Cr hybrid lies in their functional synergy rather than simple addition of reinforcements. WC provides load-bearing hardness and abrasion resistance, Cr improves interfacial bonding and stabilizes the oxide tribolayer, while WSSCs act as a ductile metallic phase offering work-hardening and crack-bridging. This multi-scale interaction shifts wear from severe adhesion to controlled micro-abrasion and simultaneously utilizes recycled material, making the system mechanistically and materially new rather than a routine compositional variation.

## Materials and methodology

### Selection of materials

To develop the desired hybrid composites based on copper matrix, commercial copper was selected and procured as the matrix due to its better ductility, resistance against corrosion, thermal conductivity, and poor hardness, tensile, and tribological behaviours^1,2^. However, waste stainless steel chips (WSSCs, due to high availability), tungsten carbide (WC), and chromium (Cr) were considered as reinforcing agents due to their good mechanical as well as tribological properties^3,4^. So, to improve the mechanical and tribological aspects of the copper metal matrix, the above-mentioned reinforcements are selected for the same. The weight percentages of the added reinforcements (WSSCs, WC, and Cr) and their variations in the copper matrix, including their weights, are reported precisely in Table 1.

**Table 1:** The details of the constituents for the developed hybrid composites using the stir-casting technique and their designation**.**

S. No	Developed materials	Designation of developed materials	Details of composition	Wt% taken of composition	Wt. taken of composition (g)
1	Cast commercial copper	CCC	Commercial Copper	100	1000
			WC	0	0
			Cr	0	0
			WSSCs	0	0
2	Cu-1WC-2Cr-1WSSCs	HC-WSSC1	Commercial Copper	96	960
			WC	1	10
			Cr	2	20
			WSSCs	1	10
3	Cu-1WC-2Cr-2WSSCs	HC-WSSC2	Commercial Copper	95	950
			WC	1	10
			Cr	2	20
			WSSCs	2	20
4	Cu-1WC-2Cr-3WSSCs	HC-WSSC3	Commercial Copper	94	940
			WC	1	10
			Cr	2	20
			WSSCs	3	30
5	Cu-1WC-2Cr-4WSSCs	HC-WSSC4	Commercial Copper	93	930
			WC	1	10
			Cr	2	20
			WSSCs	4	40

### Development of materials

**Fig. 1:**
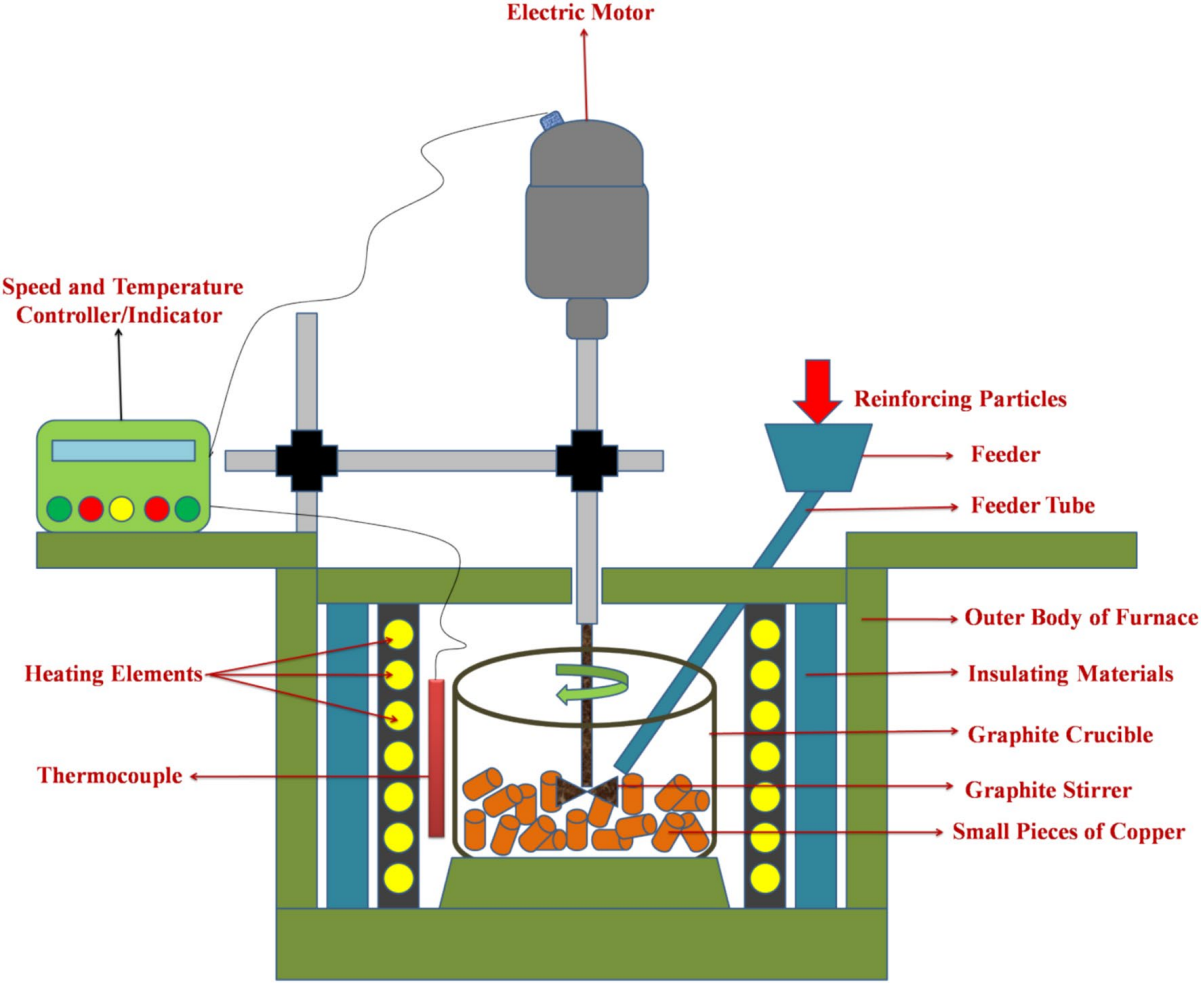
Represented the stir-casting schematic diagram to fabricate the hybrid copper composites.

Here, the stir-casting process is utilized for the development of all copper-based hybrid composites; the schematic diagram of the stir-casting technique is represented in Fig. 1. In the stir-casting process, the commercial copper metal pieces (as per the weight percentage mentioned in the Table 1) were first loaded into a crucible of graphite and put in a standing muffle furnace equipped with a microprocessor-based PID controller, which was set to the temperature of 1200 °C and start heating with a definite heating rate of 5 °C/min (300 °C/hr.) because faster heating rate may cause oxidation and temperature overshoot. When the furnace reached to the set temperature, the copper metal was fully molten, then the preheated reinforcements (WSSCs, WC, and Cr) encased in copper foil were added into the molten metal using the feeding arrangement as shown in Fig. 1. The reinforcements were preheated using an electric oven for 1 h at 150 °C to eliminate any possible evaporative contents. The mechanical stirring was applied intermittently using a graphite stirrer for approximately 30 min with the stirrer rotational speed of 300 RPM. The WC (1 wt%) and Cr (2 wt%) contents were intentionally fixed to isolate and clearly evaluate the individual effect of WSSCs addition on the composite performance. The selected WC fraction represents an optimum load-bearing reinforcement level identified from preliminary trials and literature, where higher WC contents led to particle agglomeration and reduced ductility without proportionate wear improvement. Similarly, Cr at 2 wt% was maintained to ensure consistent solid-solution strengthening and interfacial stability across all compositions. By keeping WC and Cr constant, the study eliminates compositional interaction effects and enables a controlled, systematic assessment of WSSCs influence on density, hardness, and tribological behaviour.

### Characterizations of developed materials

#### Microstructural characterizations

**Fig. 2:**
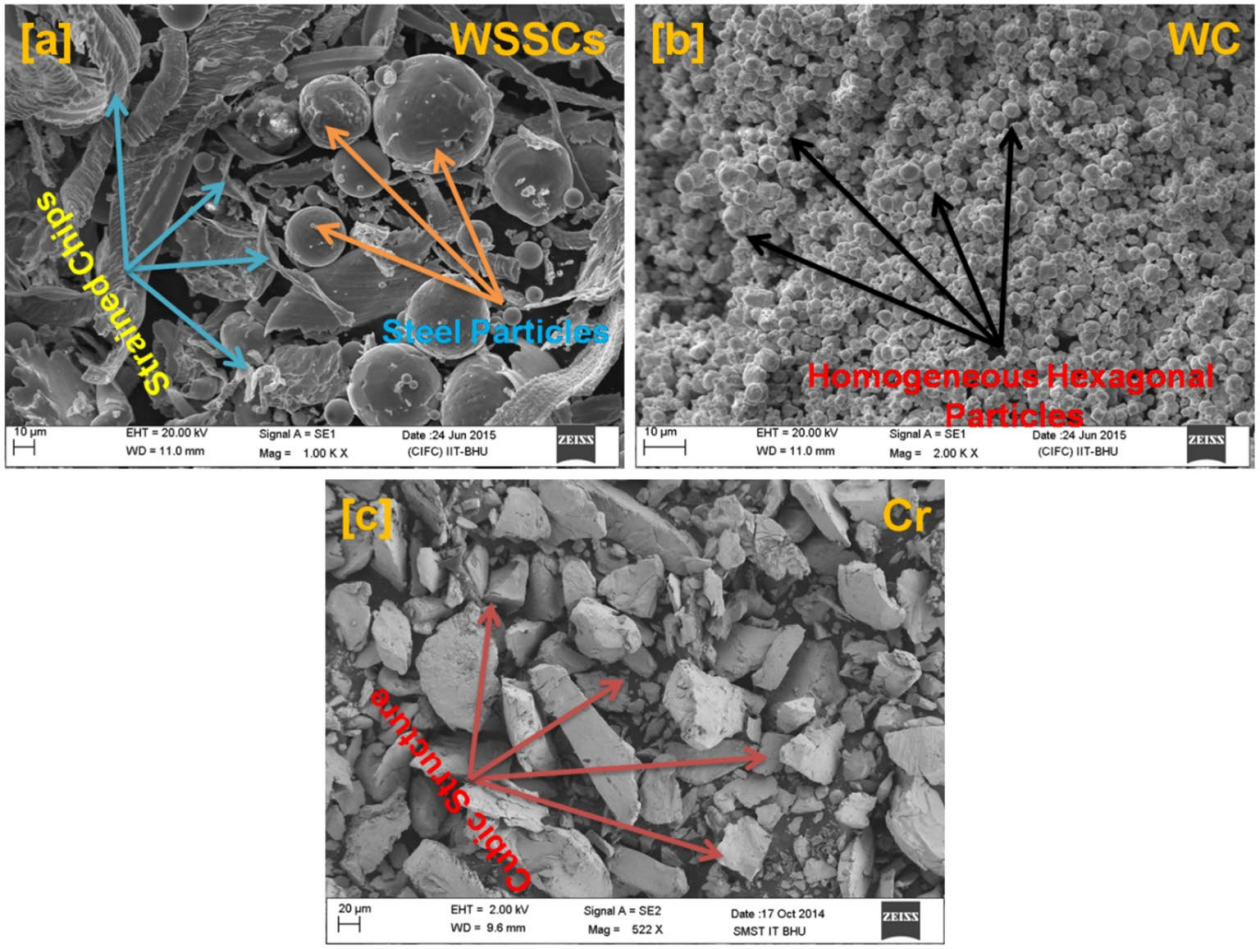
SEM micrograph of the reinforcements (**a**)WSSCs, (**b**) WC, and (**c**) Cr.

**Fig. 3:**
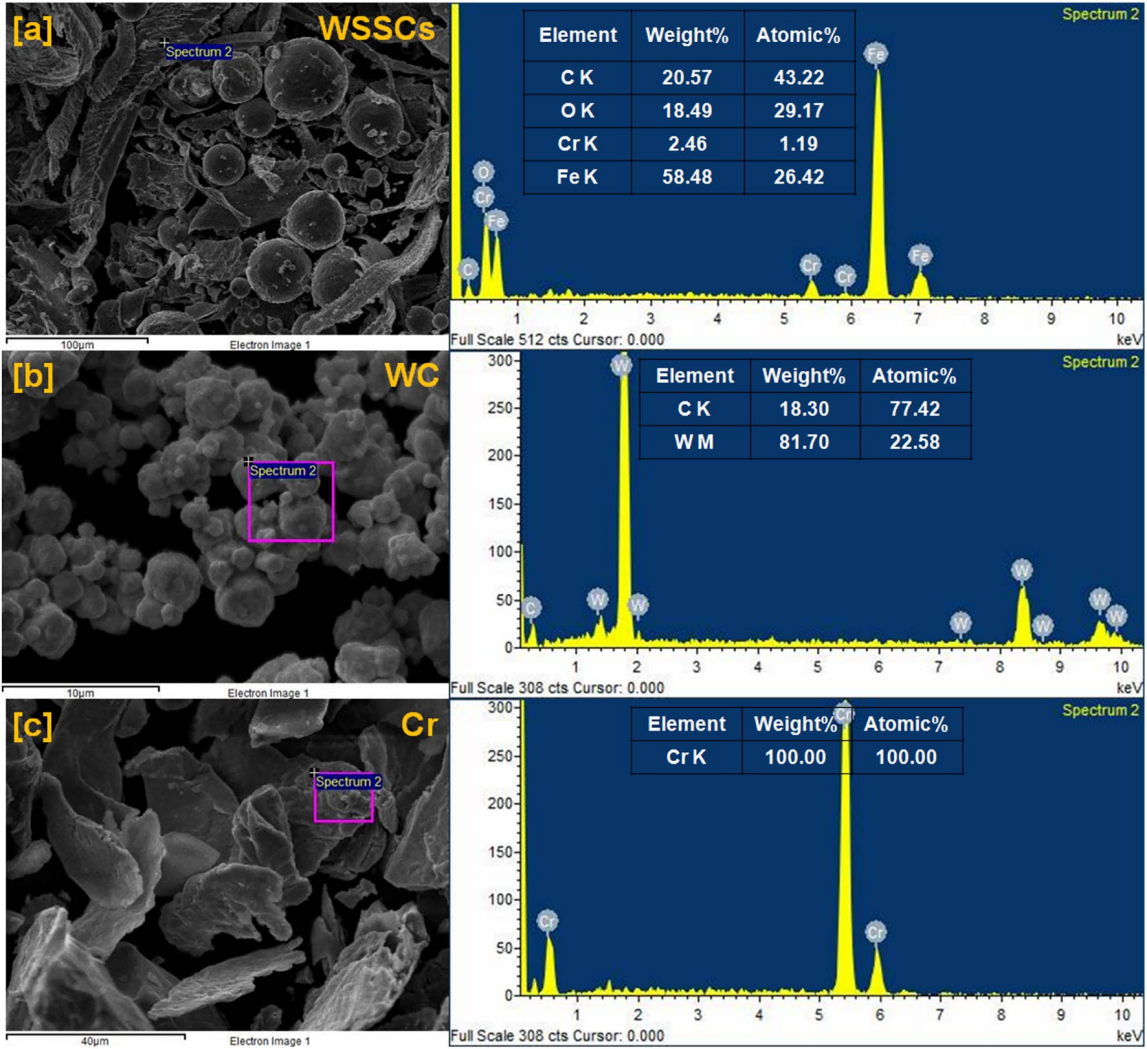
EDAX analysis of the reinforcements (**a**) WSSCs, (**b**) WC, and (**c**) Cr.

**Fig. 4:**
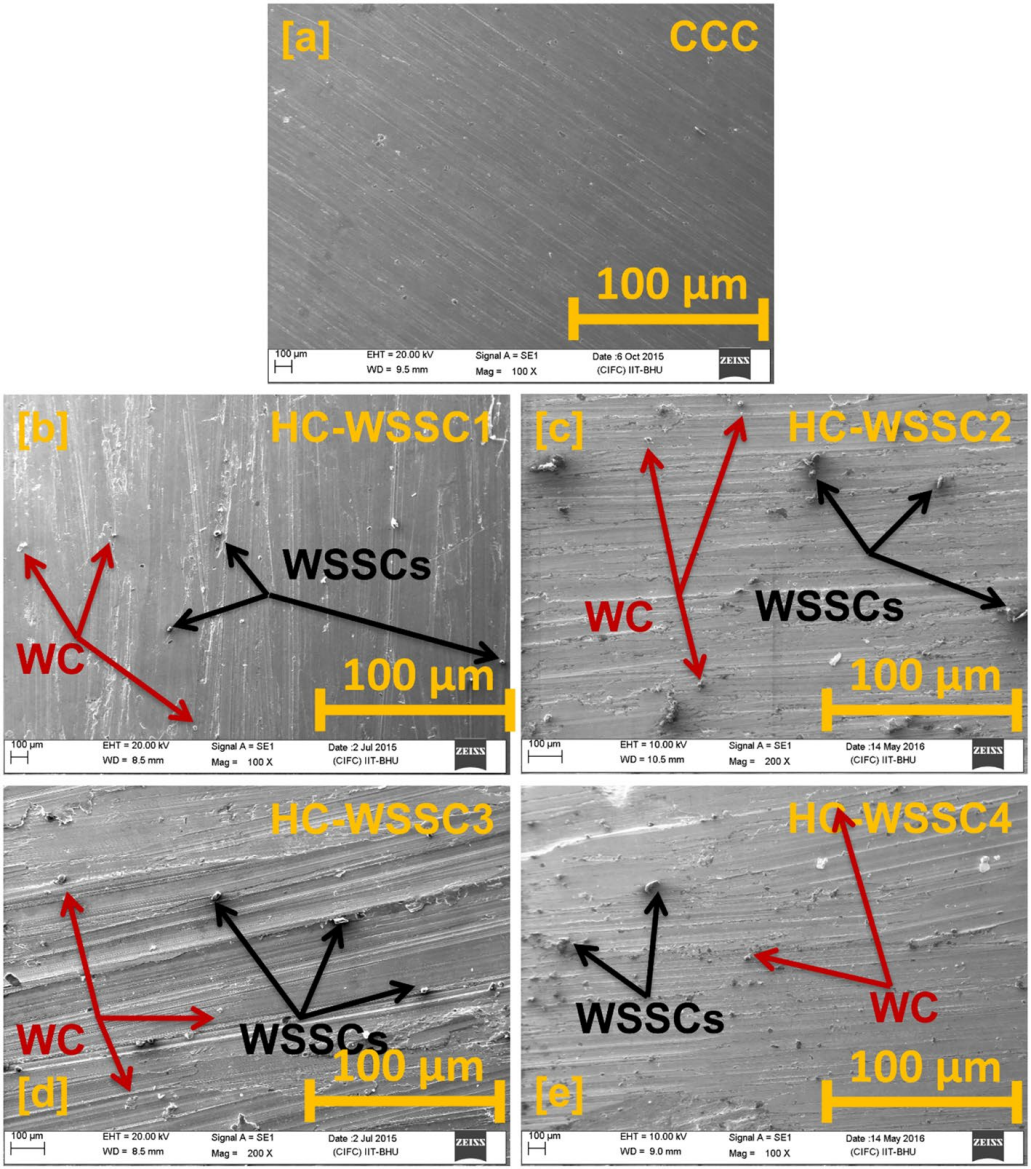
The SEM micrograph of the fabricated materials (**a**) CCC, (**b**) HC-WSSC1, (**c**) HC-WSSC2, (d) HC-WSSC3, and (**e**) HC-WSSC4.

**Fig. 5:**
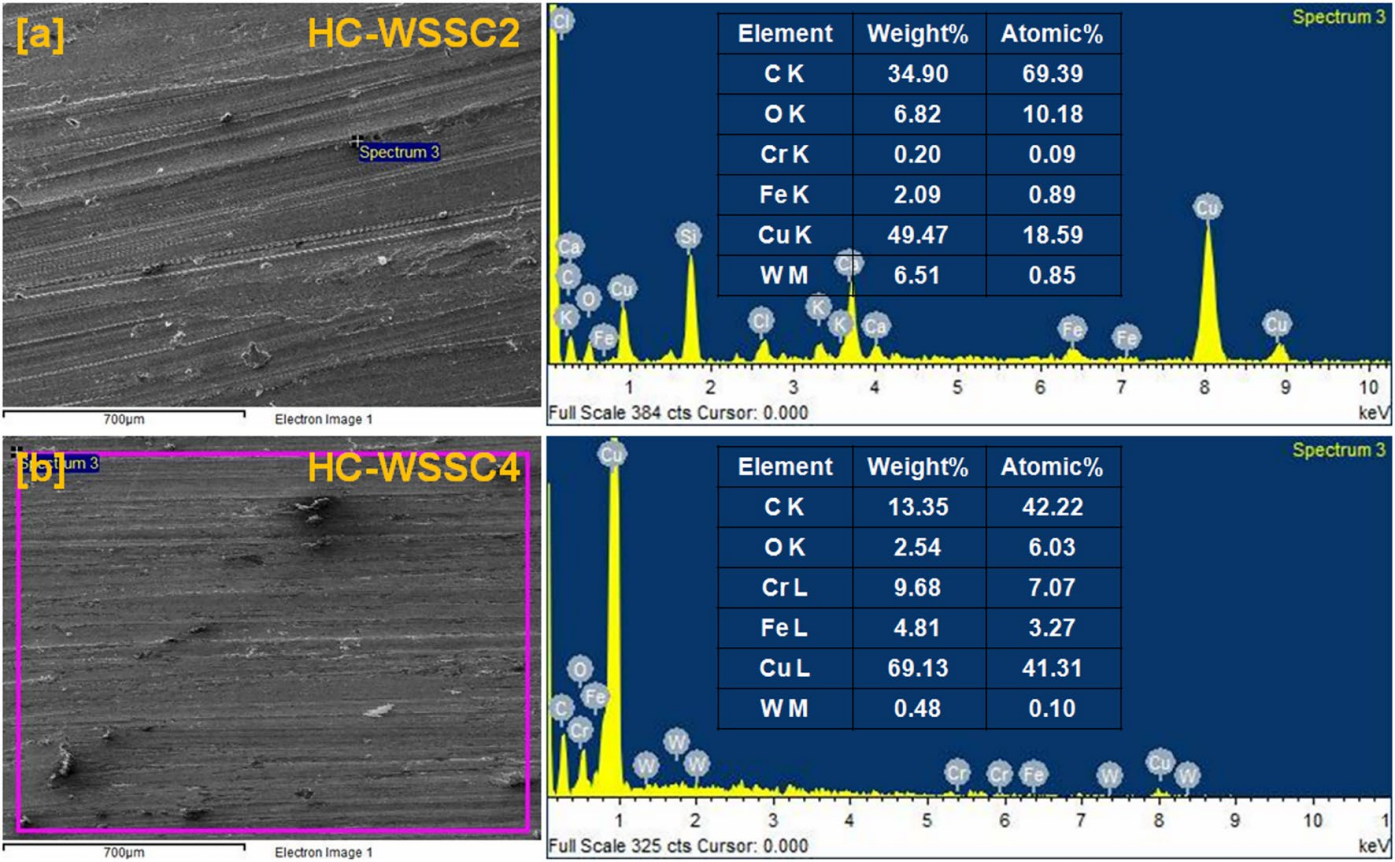
EDAX analysis of fabricated hybrid composites (**a**) HC-WSSC2 and (**b**) HC-WSSC4.

To see the size, shape, and distribution of reinforced particles in the developed hybrid composite materials with their elemental presence, the scanning electron microscope (SEM) analysis was done using NOVA Nano SEM attached with energy dispersive spectroscopy (EDS) and reported in Figs. 2, 3, 4, and 5, respectively.

#### Physical and mechanical characterizations

The experimental density (ρ_exp_) of the developed hybrid composite materials was investigated employing Archimedes principle. The requisite size of as-cast samples for testing was taken from both the end and from the mid-section of the developed sample. The Eq. (1) for density calculation is given below ^27^.

1 $$\rho = \frac{{W_{a} }}{{W_{a} - W_{w} }}$$

where ${\uprho }$—Experimental density in g/cm^3^. $W_{a}$—Weight of material in air in ‘g’. $W_{w}$—Weight of material in water in ′g′

Theoretical density (ρ_th_) of the developed materials is also evaluated to make a comparative analysis with the experimental density and to reveal the porosity developed in the materials. Theoretical density calculated using the rule of mixture formula, as shown in Eq. 2. However, the porosity is evaluated using the formula, as shown in Eq. 3. Both equations are given below to evaluate the experimental density and porosity ^28,29^.

2 $$\rho_{th} = \frac{1}{{\left( {\frac{Wm}{{\rho m}} + \frac{Wr1}{{\rho r1}} + \frac{Wr2}{{\rho r2}} + \cdots } \right)}}$$

where *ρ*_*th*_—Theoretical density g/cm^3^, _*Wm*_ $-$ Weight fraction of matrix, *W*_*r1*_— Weight fraction of reinforcement1, *W*_*r2*_–Weight fraction of reinforcement2, and so on. *ρ*_*m*_—Theoretical density of matrix, *ρ*_*r1*_—Theoretical density of reinforcement1, *ρ*_*r2*_—Theoretical density of reinforcement2 and so on.

Once theoretical density is known:

3 $$\% {\mathrm{Porosity}} = \left( {\frac{{\rho_{th} - \rho_{exp} }}{{\rho_{th} }}} \right) \times 100$$

where $\rho_{th}$ = Theoretical density g/cm^3^. $\rho_{exp}$ = Experimental density, g/cm^3^.

To evaluate the influence of reinforcement particles on material hardness, macro-hardness measurements were performed using an ATI LD3000 Brinell hardness testing machine. Specimen testing and preparation procedures were conducted as per ASTM E10 standards. To ensure data reliability and consistency, three specimens were selected from different regions, and eight indentations were made on each specimen. The hardness values reported for both the base matrix and the developed hybrid composites represent the average of all recorded measurements.

#### Friction and wear test under dry sliding condition

The friction and wear behaviour of the copper matrix and the fabricated hybrid composites was investigated at room temperature under dry sliding conditions. Friction and wear investigations were performed using a pin-on-disc (PoD) tribometer (Ducom), following the ASTM G99-17 standard. Test wear pins (specimens) were selected from the mid-section of the as-cast samples. The cylindrical pins with dimensions of 30 mm in length and 8 mm in diameter were machined using a lathe. An EN31 steel disc hardened to 60–62 HRC was used as the counterface material. Prior to testing, the samples were sequentially polished using emery papers of varying grit sizes, followed by cloth polishing. The polished specimens possessed an average surface roughness of 180 ± 10 nm. To ensure proper load-bearing contact, initial flattening of the pin surface was performed by placing 2500-grit emery paper on the disc and running the test at low load and sliding speed. Both the pin and the counter disc were thoroughly cleaned with acetone before and after each experiment. The mass of the pin specimens was recorded using a METTLER TOLEDO balance with a resolution of ± 0.1 mg.

Wear and friction tests were carried out for all the developed materials under applied normal loads ranging from 10 to 40 N, with a approximately maximum sliding distance of 5000 m at a constant rotational speed of 500 rpm. The testing conditions for the friction and wear test are precisely shown in the Table 2. Material loss was determined by measuring the mass difference of the pin before and after testing, while the coefficient of friction was continuously recorded using the data acquisition system integrated with the tribometer. The wear results are presented as cumulative weight loss (g) as a function of sliding distance. Each experimental condition was repeated three times to ensure data reliability and repeatability. To elucidate the underlying wear mechanisms, detailed examinations of the worn surfaces were performed using a field-emission scanning electron microscope (FESEM, Nova Nano SEM 450) equipped with energy-dispersive spectroscopy (EDS). Additionally, atomic force microscopy (AFM) analysis was carried out using an INTEGRA Prima system to evaluate surface topographical parameters, including average roughness, root mean square roughness, peak-to-valley height, skewness, and kurtosis.

**Table 2:** Testing conditions for friction and wear test.

S. No	Parameter	Specification/Condition	Remarks/Standards
1	Test configuration	Pin-on-disc (PoD)	As per ASTM G99
2	Counterface material	EN31 steel	Hardness: 60–62 HRC
3	Counterface surface roughness	0.3–0.7 μm	Polished condition
4	Specimen geometry	Cylindrical pin	Ø 8 mm × 30 mm length
5	Applied normal load	10 N, 20 N, 30 N, and 40 N	For the study
6	Sliding speed	~ 1.31 m/s	Controlled using disc rotation
7	Sliding distance	Approximately 5000 m	Fixed test duration of 60 min
8	Track diameter	50 mm	Constant during the test
9	Sliding time	60 min	Based on the required study
10	Environment	Dry sliding	25 ± 2 °C
11	Relative humidity	40–60%	In controlled condition
12	Wear measurement	Weight loss method	Using electronic balance (± 0.1 mg accuracy)
13	Coefficient of friction (CoF)	Recorded at every 10 min	Using load cell sensor
14	Wear calculation	Cumulative weight loss	Calculated at every 10 min
15	Repetition	Minimum 3 trials	For repeatability and statistical reliability

## Results and discussion

### Microstructural analysis

#### SEM analysis of reinforcing particles

Figure 2a–c depicts the scanning electron microscopic micrograph of the reinforcing particles like waste stainless steel chips (WSSCs), tungsten carbide (WC), and Chromium (Cr), respectively. Figure 2a clearly shows the spherical as well as strained majority chips morphology in the SEM micrograph of the WSSCs. The SEM image of WC (Fig. 2b) reveals a predominantly spherical and hexagonal particle morphology. In contrast, the SEM morphology of Cr particles appears highly irregular and rock-like in geometry, as shown in Fig. 2c. During the melting and mixing stages of the stir-casting process, these particles are likely to occupy the available spaces within the copper matrix (Fig. 1), thereby playing a significant role in enhancing the mechanical and tribological properties of the copper matrix.

#### EDAX analysis of reinforcing particles

Figure 3a–c represents the EDS spectrum analysis of the reinforcing particles like WSSCs, WC, and Cr with their corresponding SEM electron images, respectively. The EDS spectrum of the WSSCs particles, as shown in Fig. 3a reveals the presence of C K, O K, Cr K, and Fe K elements with their atomic as well as weight percentages. However, Fe K is in the major weight percentage among all. These elements suggest that the analyzed particles belong to the stainless-steel family. Figure 3b depicts the EDS spectrum of the WC particles, which shows the presence of C K and W M elements with their atomic as well as weight percentages. The major weight percentage of W M is observed. There is no other elemental peak observed in WC’s EDS spectrum, which confirms its purity at maximum. Figure 3c displays the EDS spectrum of the Cr, which does not have any elemental peak apart from the Cr K with its 100 percentage in both atomic and weight.

#### SEM analysis of fabricated materials

Figure 4a–e exhibits the SEM micrograph of stir-cast materials like CCC, HC-WSSC1, HC-WSSC2, HC-WSSC3, and HC-WSSC4, respectively. The SEM analysis is performed to determine the presence and distribution of reinforcing particles in the copper matrix. The SEM micrograph of CCC does not show any reinforcing particles because it is not reinforced, so a very smooth surface is observed. However, distributed reinforcing particles are observed in all the SEM micrographs of the casted hybrid composites (HC-WSSC1, 2, 3, and 4). The WSSCs particles are indicated using the black arrow, and WC particles are indicated using the dark red arrow in the SEM micrograph of all the composites. It is also observed that the particle distribution becomes more compact and denser as the reinforcing content of WSSCs increases from 1 to 4 wt%. Such a closer dispersion of reinforcing particles within the matrix promotes improvement in the mechanical as well as tribological properties of the developed materials^2,3,7,10^.

#### EDAX analysis of developed materials

EDS analysis of the developed composites is deliberately performed to reveal the present elements in them. In this connection, the EDS analysis of developed HC-WSSC2 and HC-WSSC4 is performed and reported, as shown in Fig. 5a, b, respectively. The elemental peaks of C K, O K, Cr K, Fe K, W M, and Cu K are observed in both the EDS spectra. This suggests that the copper-based hybrid composites have been developed successfully. EDS spectrum also suggests that as the weight percentage of WSSCs increases in the copper matrix from 2 to 4, the weight and atomic percentage of Fe K element also increases.

### Analysis of physical and mechanical behaviours

#### Density analysis of fabricated hybrid composites

**Fig. 6:**
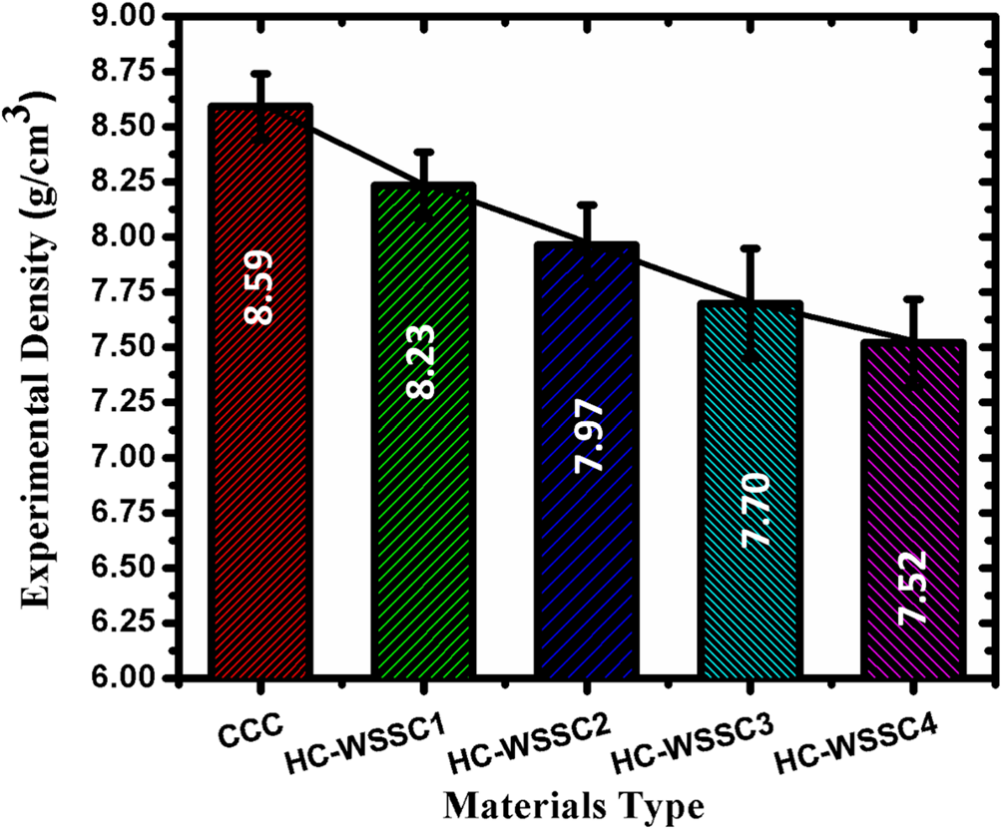
The behaviour of the experimental or measured density of the fabricated materials.

Figure 6 displays the experimental or measured density variations of the fabricated materials. A decreasing trend in the experimental or measured density of the fabricated hybrid materials is observed as the WSSCs’ weight percentage increases. The highest experimental density is observed in the copper matrix; however, HC-WSSC4 showed the least experimental density, as depicted in Fig. 6. The fabricated hybrid copper composites show experimental density lower as matched with copper metal matrix. It may be credited to the reinforcement of the theoretically lower materials’ density, like stainless steel (7.9 g/cm^3^), chromium (7.2 g/cm^3^), as compared to the density of the copper matrix (8.96 g/cm^3^). However, WC (15.6 g/cm^3^) is much heavier than copper, but its weight percentage is constant, so it may not affect or change the trend. The lowering of experimental density in hybrid composites may also be due to the development of possible oxide films on the stainless chips or on the reinforcement prevent intimate metal-particle contact and leaving micro-voids at interfaces. Lowering in experimental or measured density of hybrid copper composites may also be credited to the clustering of the reinforcing particles and thermal mismatch of the copper metal matrix and reinforcements.

The evaluated theoretical density of the developed composites shows a decreasing trend with increasing WSSC reinforcement, except for a slight initial rise, as shown in Table 3. The densities obtained for CCC, HC-WSSC1, HC-WSSC2, HC-WSSC3, and HC-WSSC4 are 8.96, 9.04, 8.86, 8.76, and 8.66 g/cm^3^, respectively. The base composite (CCC) exhibits a density close to that of pure copper because of the dominance of the Cu matrix. The marginal increase in HC-WSSC1 is attributed to the presence of relatively higher-density phases (WC and Cr) dominating the mixture at low WSSC addition. However, as the WSSC content increases from 1 to 4 wt% while WC and Cr remain constant, the overall theoretical density decreases progressively. This reduction occurs because stainless-steel chips possess lower density than copper and especially lower than WC; therefore, replacing part of the matrix volume with WSSCs reduces the average composite density according to the rule of mixtures. Consequently, the composite density gradually shifts from a Cu-rich system toward a comparatively lighter hybrid structure.

**Table 3:** A comparative detail of theoretical density, experimental density, and porosity of the developed materials.

S. No	Developed material	Theoretical density (ρ_th_), g/cm^3^	Experimental Density, (ρ_exp_), g/cm^3^	Porosity, %
1	CCC	8.96	8.59 ± 0.15	4.13
2	HC-WSSC1	9.04	8.23 ± 0.15	8.96
3	HC-WSSC1	8.86	7.97 ± 0.18	10.05
4	HC-WSSC1	8.76	7.70 ± 0.25	12.10
5	HC-WSSC1	8.66	7.52 ± 0.15	13.16

The percentage porosity of the developed composites increases progressively from 4.13% (CCC) to 8.96% (HC-WSSC1), 10.05% (HC-WSSC2), 12.10% (HC-WSSC3), and 13.16% (HC-WSSC4) as the WSSC reinforcement content increases from 1 to 4 wt%, while maintaining constant wt% of WC and Cr. This gradual rise in porosity can be attributed to the increased solid–solid interfacial area and reduced wettability between the copper matrix and WSSCs. At higher WSSC contents, particle clustering and poor interfacial bonding become more pronounced, leading to micro-void formation during compaction and sintering. Additionally, the mismatch in thermal expansion coefficients among Cu, WC, Cr, and WSSCs may induce residual stresses, further contributing to void nucleation. Consequently, the densification efficiency decreases with increasing WSSC content, resulting in higher porosity levels in the hybrid composites.

#### Hardness behaviour of the developed materials

**Fig. 7:**
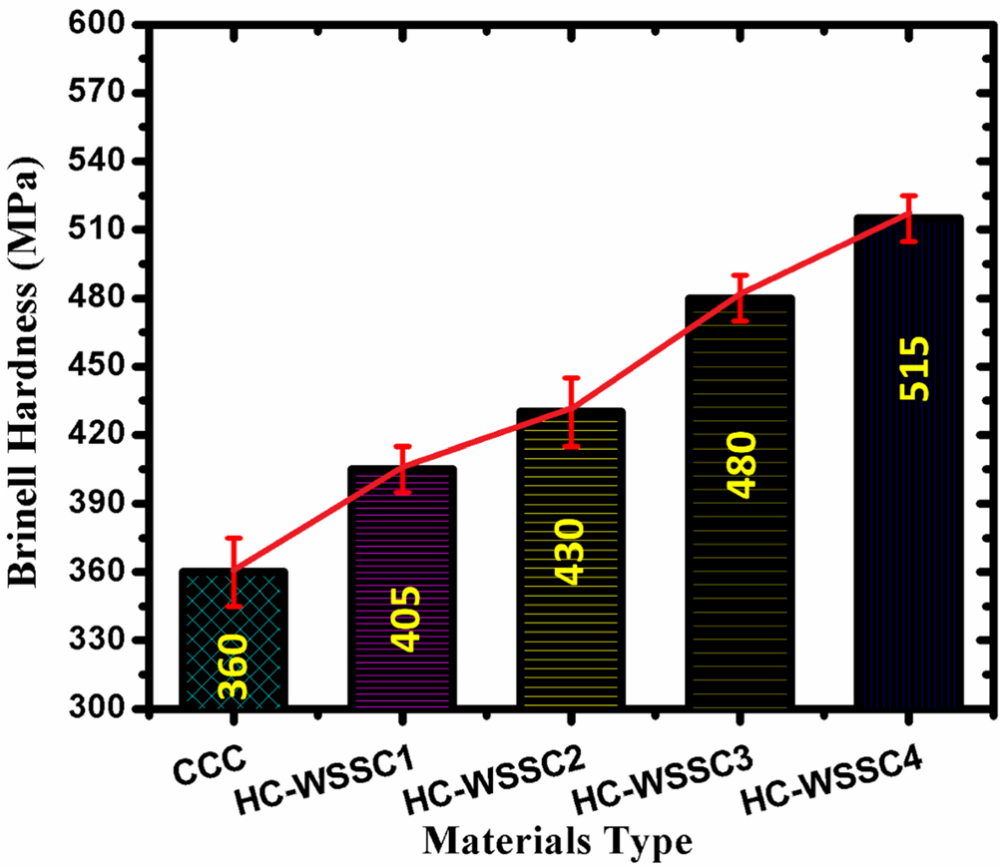
The nature of the Brinell hardness of the fabricated materials.

Figure 7 displays the nature of the Brinell hardness of the developed materials using the stir casting technique. An increasing trend of Brinell hardness is observed as the WSSCs’ weight percentage increases. Overall, the fabricated copper-based hybrid composites exhibit higher Brinell hardness than the copper metal matrix. This advancement in the hardness of hybrid copper composites may be credited to the incorporation of hard reinforcement such as WC, WSSCs, and Cr. WC, a ceramic compound, is well known for its extremely high wear resistance and hardness, contributing to the strengthening of the matrix through a dispersion strengthening mechanism. WSSCs, being metallic and relatively ductile compared to WC but harder than copper, serve as a secondary, harder reinforcement that influences the composite distinctly. The WSSCs not only increase the overall hardness when distributed optimally but also help in improving the load-carrying capability of the fabricated hybrid composites. The improvement in the hardness of composite materials may also be credited to the uniform distribution of the reinforcements within the molten copper matrix due to the stirring process. During this stirring, WSSCs, due to their size and density closer to copper, are more likely to achieve better wettability and distribution than heavier, denser WC particles. Proper wetting and interfacial connection between the reinforcements and matrix are demanding for effective load transfer and for the development of higher hardness values. From Fig. 7, the experimental Brinell hardness characteristics displayed a progressive improvement in hardness value with improving weight percentage of WSSCs, while WC and Cr contents remained constant. This trend can be attributed to WSSCs’ content, which increases the total volume of reinforcements, reducing the effective ductile matrix content. This contributes to higher resistance to indentation; more load is transferred to the harder reinforcement phases during indentation, resulting in higher hardness values. WSSCs disrupt the plastic deformation zones around the indenter, increasing resistance to penetration.

### Analysis of friction and wear behaviors

#### Friction and wear analysis

**Fig. 8:**
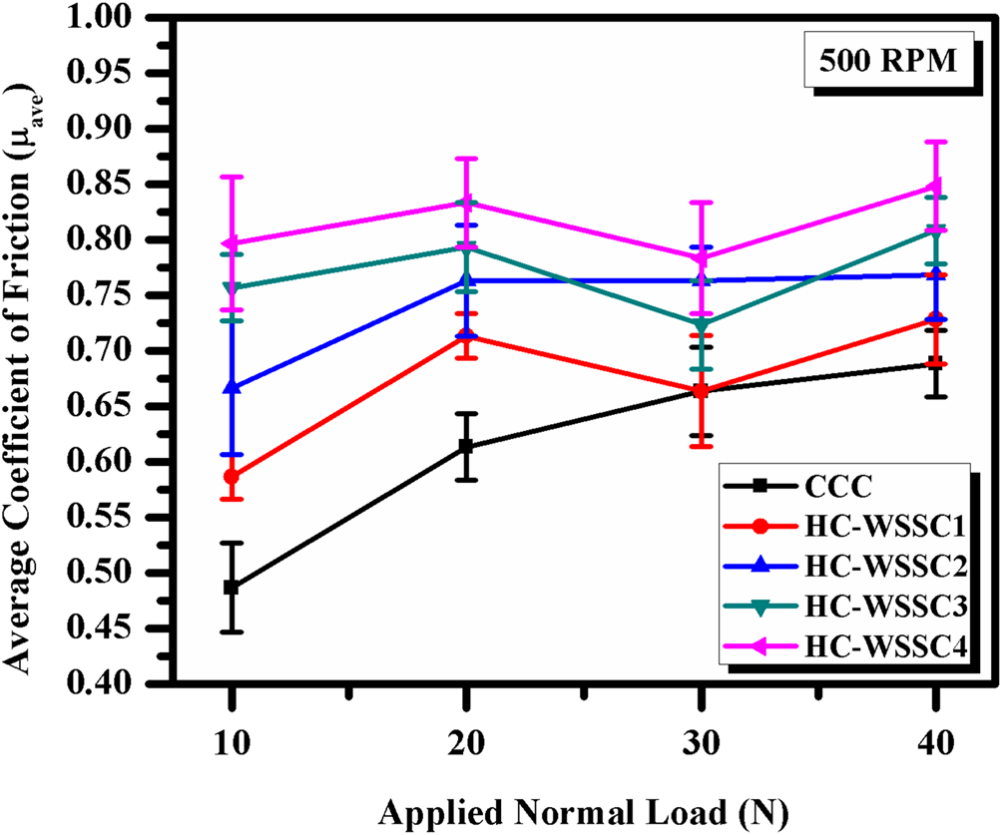
The average friction coefficient behaviour vs applied normal load for developed materials.

The deviations of the average friction coefficient with applied normal load for all developed materials using stir casting technique is shown Fig. 8. There is not a specific trend in average friction coefficient vs applied normal load observed, a fluctuating behaviour can be seen, as shown in Fig. 8. Although, all the fabricated hybrid copper composites display an enhanced average friction coefficient as matched to the copper’s average coefficient of friction. It may be attributed to the incorporation of harder reinforcements such as WSSCs, WC, and Cr into the copper metal matrix, which enhances its hardness, as discussed above. When these hard phases come into contact with the rotating EN31 steel counter-disc, the coefficient of friction increases.

**Fig. 9:**
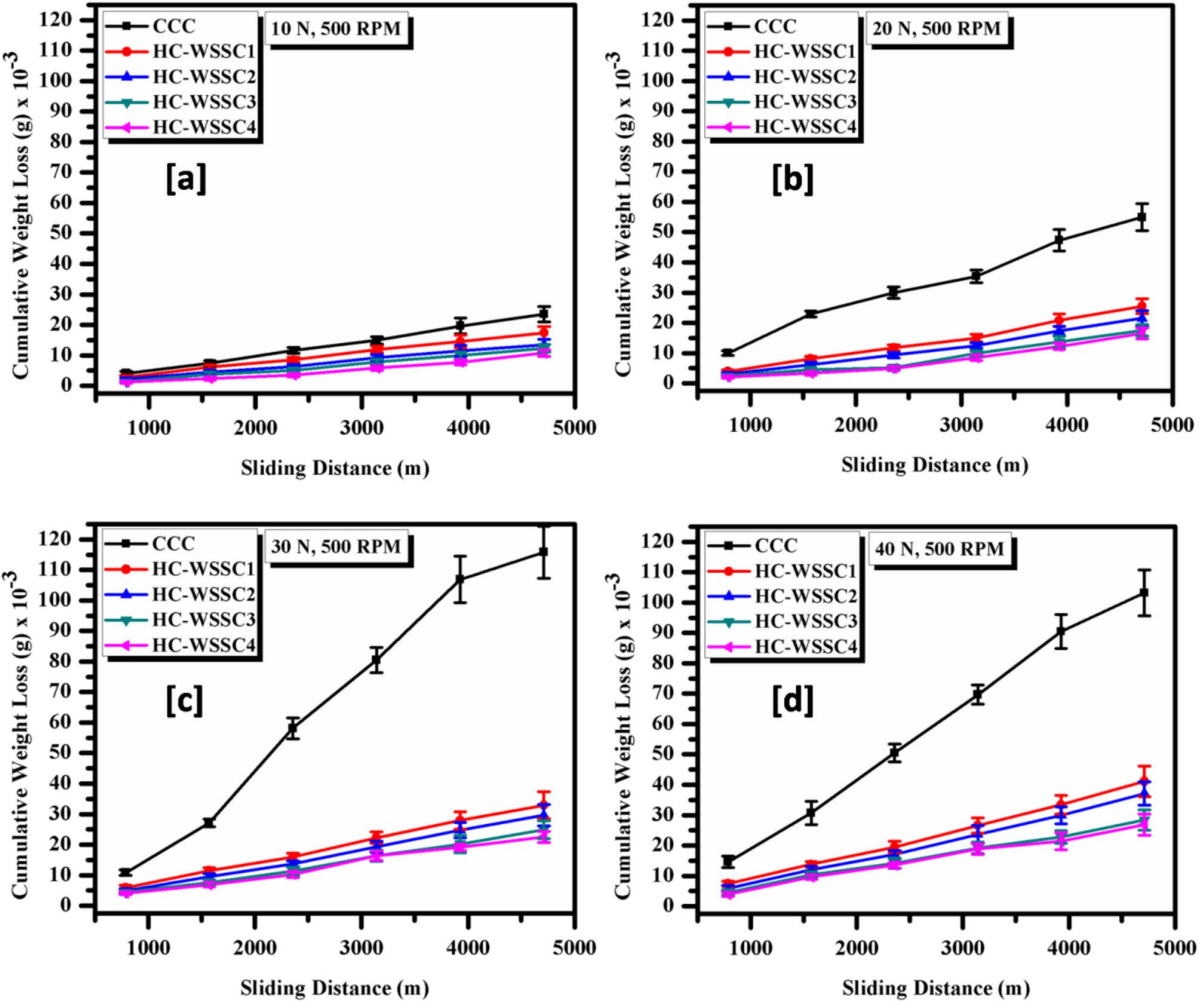
The behaviour of cumulative weight loss vs sliding distance for the fabricated materials at normal loads of (**a**) 10 N, (**b**) 20 N, (**c**) 30 N, and (**d**) 40 N.

Figure 9a–d depicts the behaviour of cumulative weight loss with respect to the sliding distance of all developed materials at a constant rpm of 500 and the applied normal loads of 10 N, 20 N, 30 N, and 40 N, respectively. Under all conditions, the fabricated hybrid copper composites show reduced cumulative weight loss, as matched with the cumulative weight loss in the copper matrix. It may be credited to the higher hardness of the developed hybrid copper composites as compared to the copper matrix, which obeys the law of wear given by Archard, in which loss of materials is inversely proportional to the materials’ hardness. Since the highest hardness was observed in HC-WSSC4, it shows the least cumulative weight loss among all the developed materials at all testing conditions. From all the figures, it is also found that cumulative weight loss increases as the applied normal load increases from 10 to 40 N for the sliding distance of approximately 5000 m at 500 rpm. The low wear loss in developed hybrid composites may also attributed to the addition of Cr in copper matrix with other reinforcements. Cr improves wettability and bonding between the Cu matrix and WSSCs particles during casting, reducing particle pull-out. During sliding, it forms a stable protective tribofilm, which suppresses adhesion and stabilizes the tribolayer. Thus, together with WC (load bearing) and WSSCs (debris accommodation), chromium enhances interfacial integrity and wear resistance beyond typical Cu-based hybrid systems.

#### Worn surface analysis using SEM

**Fig. 10:**
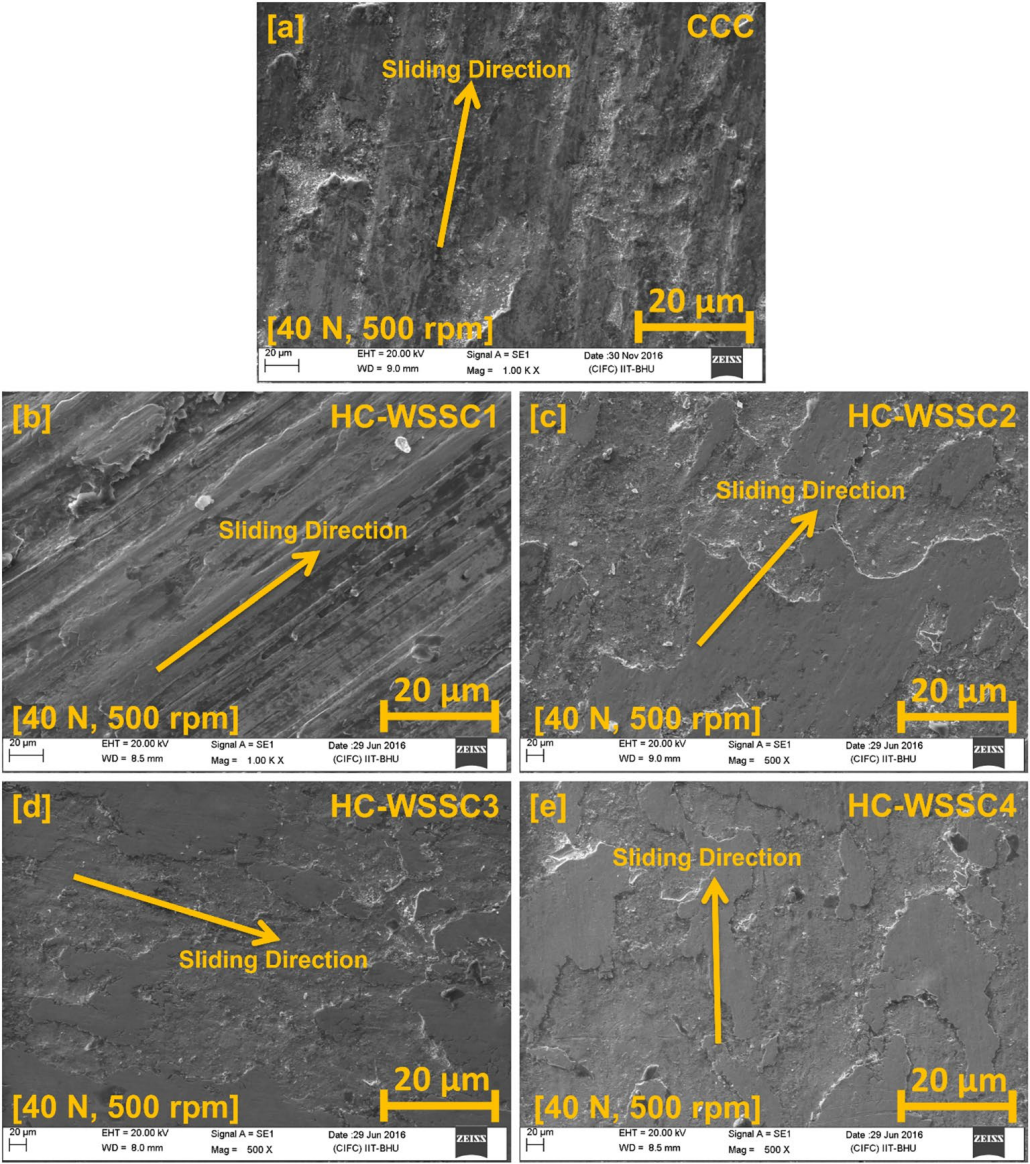
SEM images of the worn or tattered surface at 40 N and 500 rpm of the fabricated materials (**a**) CCC, (**b**) HC-WSSC1, (**c**) HC-WSSC2, (**d**) HC-WSSC3, and (**e**) HC-WSSC4.

Figure 10a–e exhibits the SEM images of the worn or tattered surface at 40 N and 500 rpm of the developed materials, such as CCC, HC-WSSC1, HC-WSSC2, HC-WSSC3, and HC-WSSC4, respectively. Sliding direction and wear track can be easily noticed in all the SEM images of the worn or tattered surfaces of all the developed materials. SEM images of the worn or tattered surfaces of HC-WSSC3 and HC-WSSC4 exhibit the less damaged surface, which suggests that low wear happened (due to their high hardness value) in these materials during the friction as well as wear test under the conditions of dry sliding^30^. SEM examination of the worn surfaces reveals well-defined wear tracks along the sliding direction, which is characteristic of abrasive and adhesive wear. The smoother and less damaged surfaces observed in HC-WSSC3 and HC-WSSC4 further confirm reduced material detachment and stable sliding conditions due to improved hardness and reinforcement distribution.

#### Worn surface analysis using EDAX

**Fig. 11:**
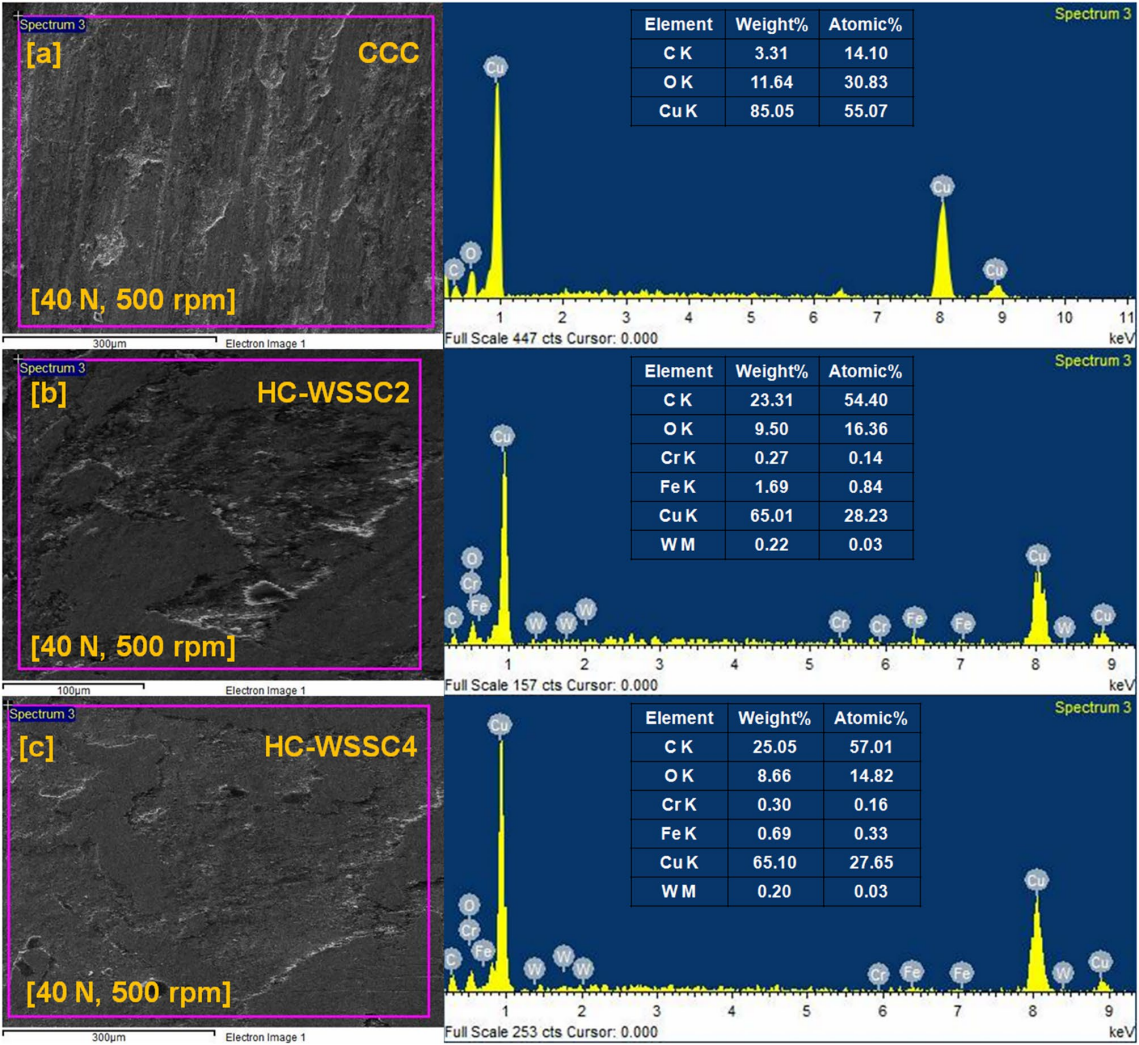
The EDAX analysis of the worn or tattered surface at 40 N and 500 rpm of the fabricated materials (**a**) CCC, (**b**) HC-WSSC2, and (**c**) HC-WSSC4.

The EDAX or EDS analysis was also executed to know the wear mechanism involved during the dry sliding test of all the materials using stir casting. Figure 11a–c depicts the EDS spectrum of CCC, HC-WSSC2, and HC-WSSC4 with their corresponding selected area of SEM micrographs, respectively, at an applied normal load of 40 N and 500 rpm. Elements C K, O K, and Cu K are observed with their atomic as well as weight percentage in the EDS spectrum of the CCC, as shown in Fig. 11a. It suggests that during the dry sliding of CCC, there is a possible development of the oxide of the metal due to an increase in interface temperature at a higher applied normal load of 40 N^31^. The same reason is valid for the presence of the O K element in the EDS spectrum of HC-WSSC3 and HC-WSSC4. Additionally, the presence of oxygen (O K) peaks in the EDS spectra of copper and hybrid composites indicates the formation of oxide layers during dry sliding, particularly at higher loads. This points to the occurrence of oxidative wear, resulting from elevated interfacial temperatures generated during sliding. The formation of a mechanically mixed oxide layer may act as a protective tribofilm, contributing to reduced wear in harder hybrid composites.

#### Worn surface analysis using AFM

**Fig. 12:**
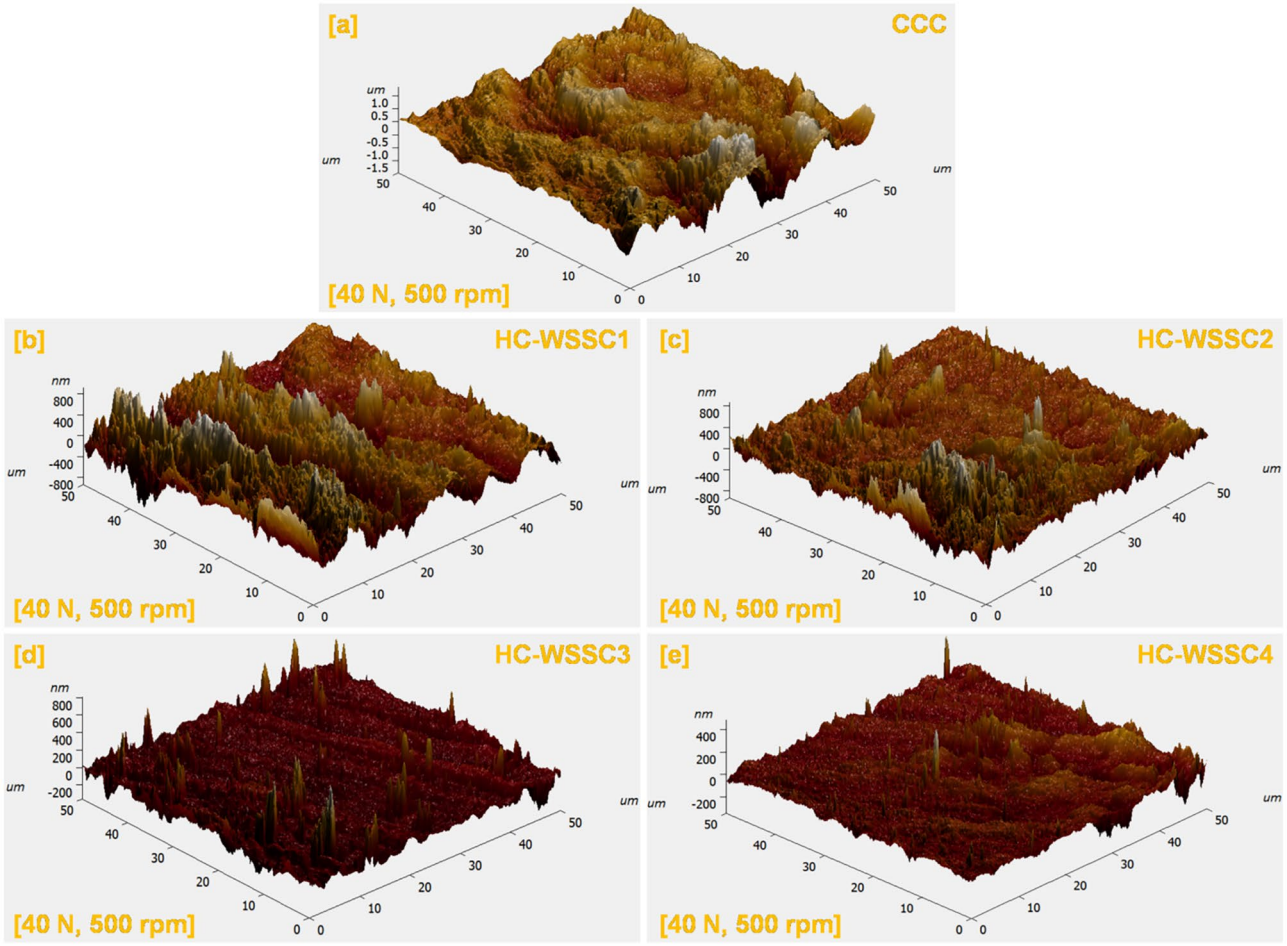
AFM micrograph of the dry sliding wear-tested specimen at normal load of 40 N and 500 rpm of the fabricated materials (**a**) CCC, (**b**) HC-WSSC1, (**c**) HC-WSSC2, (**d**) HC-WSSC3, and (**e**) HC-WSSC4.

The atomic force microscopy (AFM) is highly effective for accurately quantifying nano-metric surface roughness, enabling a detailed evaluation of topographical appearance. A 50 micron^2^ area was preferred to assess the surface topography or profile of the matrix and the developed or fabricated composites. Three-dimensional AFM micrographs of the worn surfaces were captured under identical testing circumstances: a 40 N load and 500 rpm. This test was conducted for both the matrix and developed composites under dry sliding conditions, and their morphologies are depicted in Fig. 12. The worn surfaces of both materials show undulating appearances resembling ups and downs, indicating a wavy texture, which is precisely exhibited in Fig. 12a–e. For the matrix CCC, the maximum top-to-bottom height is close to 353.7 nm under dry sliding conditions. In contrast, the developed composites reinforced with WSSC show a significantly lower surface roughness as compared to CCC’s roughness, with peak-to-valley heights of approximately 127.7 nm, 117.6 nm, 114.1 nm, and 45.0 nm for HC-WSSC1, HC-WSSC2, HC-WSSC3, and HC-WSSC4, respectively, under dry sliding conditions. These findings suggest that composite experiences notably less surface damage and, consequently, reduced wear. Although AFM indicates smoother surfaces (lower Ra) at higher WSSC content, friction can increase due to changes in interfacial shear behavior rather than surface topography alone. The addition of WSSCs increases surface hardness and real contact stability, which reduces plastic smoothing but promotes stronger asperity interlocking and higher shear strength at the interface. Moreover, the formation of hard tribo-oxide layers and exposed stainless-steel phases increases interfacial adhesion and ploughing resistance. Thus, despite reduced roughness, the elevated interfacial shear strength and load-bearing rigidity lead to higher friction coefficients.

Worn or tattered surface roughness parameters of the investigated specimen were examined using the AFM (Atomic Force Microscopy) to reveal the clear surface textures of the worn or tattered surfaces, and their values are reported in Table 4. Surface morphology is a factor that significantly affects the friction and wear responses of the materials. These parameters contain the peak or crest to basin or valley height, known as R_t_, the average or mean surface roughness or R_a_, the root mean square roughness (R_q_), kurtosis (R_ku_), and skewness (R_sk_). The parameter R_a_, obtained from the AFM examinations, reveals the average surface height over a chosen area and is commonly used to describe the roughness of worn surfaces of investigated materials. It is especially useful for identifying changes in surface texture and for monitoring the consistency of the processes. R_t_ reflects the total vertical distance between the highest peak and the lowest valley within the measured area, thereby characterizing the overall surface roughness of the investigated materials. R_q_ quantifies the standard deviation of surface heights, offering more precise insight than R_a_, particularly when there are significant deviations from the mean plane. It also plays a key role in calculating R_sk_ and R_ku_^32,33^.

**Table 4:** Worn surface roughness parameters of the investigated specimen under dry sliding conditions.

S. No	Developed materials	Roughness values of worn surfaces of developed materials					
		Ra (nm)	Rq (nm)	Rt (nm)	Rq/Ra	Rsk	Rku
1	CCC	195	235	353.7	1.21	0.727	5.669
2	HC-WSSC1	194	244	127.7	1.26	0.631	4.397
3	HC-WSSC2	123	186	117.6	1.51	− 0.198	3.341
4	HC-WSSC3	45.7	60.6	114.1	1.33	− 0.182	3.124
5	HC-WSSC4	34.1	44.9	45.0	1.32	− 0.0619	2.912

R_sk_ measures the asymmetry of the worn surface profile about the mean plane. It is sensitive to isolated sharp peaks or deep valleys and is useful for evaluating properties such as load-bearing capacity, porosity, and the nature of non-conventional wear mechanisms. A negative R_sk_ indicates a surface with dominant valleys, typically associated with better load-bearing capabilities. This parameter is often used to distinguish between surfaces that may have the same R_a_ or R_q_ but differ in profile shape. R_ku_ provides information on the sharpness or flatness of the surface profile by evaluating the distribution of height extremes. It is particularly useful in understanding the structure of worn layers and is frequently referenced for stress fracture analysis. An R_ku_ value greater than 3 suggests a spiky surface, while a value less than 3 demonstrates a wavier surface morphology. A 3 value exactly reflects a perfectly random surface distribution.

As reported in Table 4, R_a_ significantly decreases from 195 to 34.1 nm under the conditions of dry sliding as the reinforcement content of WSSC increases from 0 to 4 wt. %. Notably, both the matrix and developed hybrid composites exhibit a higher R_q_ value than R_a_ under dry sliding conditions^34^. According to statistical theory, in a Gaussian distribution of roughness or asperity heights, the R_q_/R_a_ ratio should be close to 1.25. From the investigation, it is found that for most worn surfaces, this ratio can be as high as 1.31 and still be well represented using a Gaussian distribution. The R_q_/R_a_ values obtained from AFM scans of the worn or tattered surfaces (for matrix and developed hybrid composites, both under dry sliding conditions) closely match the theoretical value of 1.25, as detailed shown in Table 4. It indicates that, on a macroscopic scale, the peak distribution of asperities on the worn or tattered surfaces resembles a Gaussian pattern, thereby validating the use of statistical models to describe the worn surface roughness.

Moreover, the negative R_sk_ values observed for the worn surfaces of developed composites suggest that valleys dominate the surface topography. In contrast, the positive R_sk_ values for the CCC and HC-WSSC1 imply that peaks are more prevalent. Negative skewness values are typically associated with valley features or cracks. The increasingly negative R_sk_ values further imply enhanced load-bearing capacity for the composite, thus broadening its applicability under high-performance operating conditions. For the developed hybrid composites except HC-WSSC1, R_ku_ values are close to 3, which indicates a relatively balanced distribution with fewer sharp peaks or deep valleys resulting in a moderately rough worn surface. In contrast, the alloy shows R_ku_ values significantly above 3, which reflects a spikier worn surface characterized by more pronounced peaks and valleys. These higher R_ku_ values correspond with higher R_t_ values, underscoring the strong correlation between worn surface peak/trough features and kurtosis. The unusually high R_q_/R_a_ ratio for HC-WSSC2 indicates non-uniform surface damage with isolated deep grooves and sharp asperities, as shown in Fig. 12c also. This likely results from local particle pull-out or micro-fracture of reinforcement clusters, which raises R_q_ more than Ra and signifies intermittent abrasive micro-cutting during sliding.

Table 4 shows the roughness values of worn surfaces of the developed materials during the wear test at 500 rpm, 40 N load for 60 min, the roughness parameters R_a_, R_sk_, and R_ku_ clearly indicate the active wear mechanisms in the CCC, HC-WSSC1, HC-WSSC2, HC-WSSC3, and HC-WSSC4 composites. Ra represents overall surface damage; higher values correspond to severe adhesive/abrasive wear, while lower Ra indicates improved wear resistance due to load-bearing WSSCs, WC particles and stabilizing Cr phases forming a protective tribolayer. R_sk_ (skewness) describes surface profile nature: negative R_sk_ indicates valley-dominated surfaces associated with micro-ploughing and controlled abrasion, whereas positive Rsk suggests peak-dominated surfaces typical of adhesive material transfer. R_ku_ (kurtosis) reflects asperity sharpness: R_ku_ > 3 indicates micro-cutting abrasion, R_ku_ ≈ 3 uniform wear, and R_ku_ < 3 plastic smearing due to adhesion. Overall, the combined trend of reduced Ra with slightly negative R_sk_ and moderate R_ku_ signifies a transition from adhesive wear in the Cu-rich composite to controlled abrasive wear in the reinforced hybrids.

Valley-dominated surfaces (negative R_sk_) improve load-bearing during sliding by allowing flattened asperity plateaus to support the applied load, while valleys trap wear debris and retain tribofilms. This reduces stress concentration, minimizes third-body abrasion, and stabilizes the contact interface, thereby enhancing load distribution and wear resistance.

#### Correlation behaviour of hardness and wear loss analysis

**Fig. 13:**
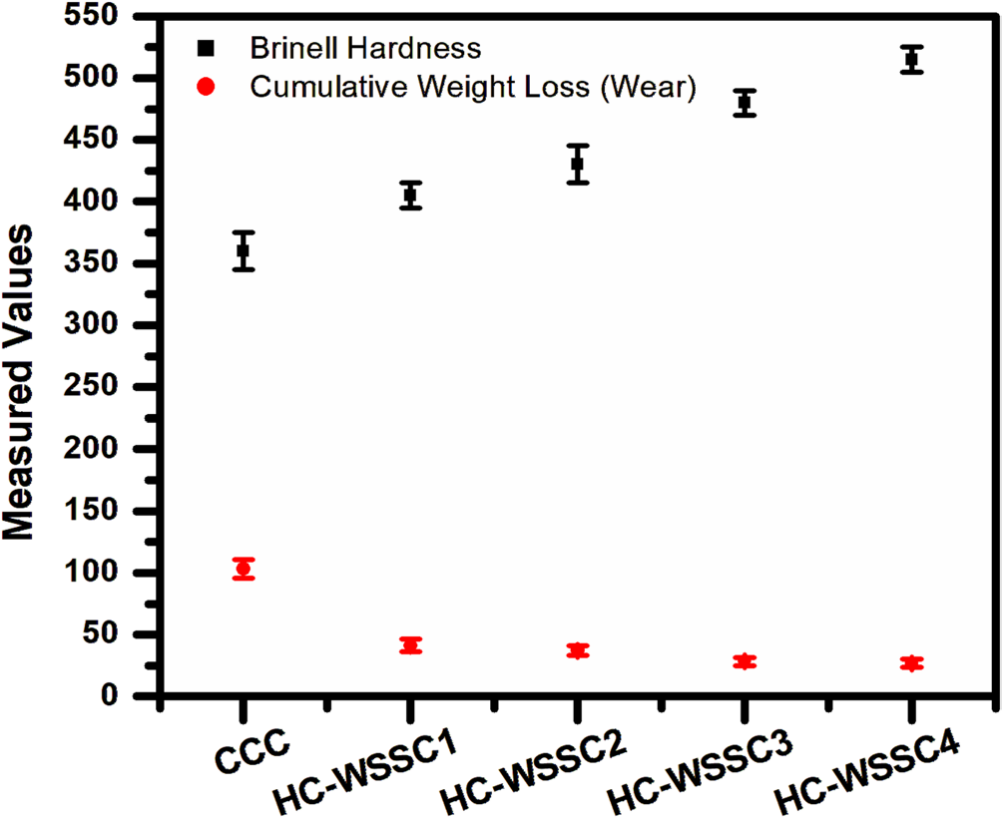
The correlation behaviour of hardness and wear loss (cumulative weight loss) of developed materials.

Figure 13 exhibits the correlation behaviour of hardness and wear loss for the developed materials (CCC, HC-WSSC1, HC-WSSC2, HC-WSSC3, and HC-WSSC4). It is observed that as the hardness of the materials increases the wear loss (cumulative weight loss) decreases. This observed decrease in wear loss with increasing hardness is attributed to improved resistance to plastic deformation at the sliding interface. Higher hardness enhances the load-bearing capacity of the surface and suppresses asperity penetration, thereby reducing adhesive junction formation and micro-ploughing. Hard reinforcements act as barriers to dislocation motion and limit material removal, while also promoting the formation of a stable tribolayer. Consequently, the real contact area and subsurface shear deformation decrease, leading to lower wear volume in accordance with Archard’s wear relationship, where wear loss is inversely proportional to hardness.

## Conclusion

After analyzing the complete research work, the following conclusions which are reported as follows:

- Copper-based hybrid composites were fabricated by stir casting with varying WSSC content while keeping WC and Cr constant. SEM and EDAX/EDS analyses confirmed uniform reinforcement dispersion and good interfacial bonding within the copper matrix.
- Developed hybrid composites revealed good mechanical (Brinell Hardness) as well as tribological (friction and wear) behaviors. The developed hybrid composites such as HC-WSSC1, HC-WSSC2, HC-WSSC3, HC-WSSC4 have shown improved hardness of 12.5%, 19.44%, 33.33%, and 43.06%, respectively, as compared to the copper matrix (CCC).
- The evaluated theoretical density of the developed composites shows a decreasing trend with increasing WSSC reinforcement, except for a slight initial rise, as shown in Table 3. The densities obtained for CCC, HC-WSSC1, HC-WSSC2, HC-WSSC3, and HC-WSSC4 are 8.96, 9.04, 8.86, 8.76, and 8.66 g/cm^3^, respectively.
- A reduction in the measured experimental density of 4.19%, 7.22%, 10.36%, and 12.46% was noted in the fabricated hybrid composites such as HC-WSSC1, HC-WSSC2, HC-WSSC3, HC-WSSC4, respectively when compared with the monolithic copper matrix (CCC), while maintaining the same overall material characteristics.
- The percentage porosity of the developed composites increases progressively from 4.13% (CCC) to 8.96% (HC-WSSC1), 10.05% (HC-WSSC2), 12.10% (HC-WSSC3), and 13.16% (HC-WSSC4) as the WSSC reinforcement content increases from 1 to 4 wt%.
- From the SEM and EDAX/EDS analysis, the dominant wear mechanisms involved in the present study include abrasive wear, mild adhesive wear, and oxidative wear, with their relative contributions governed by applied load, hardness, and reinforcement content of the developed hybrid copper composites.
- AFM analysis revealed a significant improvement in worn surface quality with increasing WSSC reinforcement, as evidenced by a substantial reduction in surface roughness (Ra) from 195 to 34.1 nm, indicating enhanced wear resistance and smoother tribological contact in the developed hybrid composites.
- The negative skewness (Rsk) and near-Gaussian roughness distribution observed in higher WSSC-content composites confirm valley-dominated, load-bearing surfaces with stable wear behaviour, while Rku values close to 3 suggest a balanced surface morphology suitable for high-performance sliding applications.

## Data Availability

Data is made available on the request.
